# Using *in Vitro* High Throughput Screening Assays to Identify Potential Endocrine-Disrupting Chemicals

**DOI:** 10.1289/ehp.1205065

**Published:** 2012-09-28

**Authors:** Daniel M. Rotroff, David J. Dix, Keith A. Houck, Thomas B. Knudsen, Matthew T. Martin, Keith W. McLaurin, David M. Reif, Kevin M. Crofton, Amar V. Singh, Menghang Xia, Ruili Huang, Richard S. Judson

**Affiliations:** 1 Department of Environmental Sciences and Engineering, University of North Carolina, Chapel Hill, North Carolina, USA; 2 National Center for Computational Toxicology, and; 3 National Health and Environmental Effects Research Laboratory, Office of Research and Development, U.S. Environmental Protection Agency, Research Triangle Park, North Carolina, USA; 4 Lockheed Martin, Research Triangle Park, North Carolina, USA; 5 National Center for Advancing Translational Sciences, National Institutes of Health, Department of Health and Human Services, Bethesda, Maryland, USA

**Keywords:** androgen, endocrine, estrogen, high throughput, *in vitro*, ToxCast

## Abstract

Background: Over the past 20 years, an increased focus on detecting environmental chemicals that pose a risk of adverse effects due to endocrine disruption has driven the creation of the U.S. Environmental Protection Agency (EPA) Endocrine Disruptor Screening Program (EDSP). Thousands of chemicals are subject to the EDSP; thus, processing these chemicals using current test batteries could require millions of dollars and decades. A need for increased throughput and efficiency motivated the development of methods using *in vitro* high throughput screening (HTS) assays to prioritize chemicals for EDSP Tier 1 screening (T1S).

Objective: In this study we used U.S. EPA ToxCast HTS assays for estrogen, androgen, steroidogenic, and thyroid-disrupting mechanisms to classify compounds and compare ToxCast results to *in vitro* and *in vivo* data from EDSP T1S assays.

Method: We implemented an iterative model that optimized the ability of endocrine-related HTS assays to predict components of EDSP T1S and related results. Balanced accuracy was used as a measure of model performance.

Results: ToxCast estrogen receptor and androgen receptor assays predicted the results of relevant EDSP T1S assays with balanced accuracies of 0.91 (*p* < 0.001) and 0.92 (*p* < 0.001), respectively. Uterotrophic and Hershberger assay results were predicted with balanced accuracies of 0.89 (*p* < 0.001) and 1 (*p* < 0.001), respectively. Models for steroidogenic and thyroid-related effects could not be developed with the currently published ToxCast data.

Conclusions: Overall, results suggest that current ToxCast assays can accurately identify chemicals with potential to interact with the estrogenic and androgenic pathways, and could help prioritize chemicals for EDSP T1S assays.

Endocrine hormones regulate a diverse set of physiological responses, some of which include sexual dimorphism, reproductive capacity, glucose metabolism, and blood pressure (Cooper and Kavlock 1997; de Mello et al. 2011; Dupont et al. 2000; Lodish et al. 2009; Ng et al. 2001). The many types of responses regulated by hormones makes them of particular concern for disruption by xenobiotics (Ankley and Giesy 1998; Colborn and Clement 1992; Soto and Sonnenschein 2010; Tilghman et al. 2010). Endocrine disruption can lead to many adverse consequences, some of which include altered reproductive performance and hormonally mediated cancers (Birnbaum and Fenton 2003; Kavlock et al. 1996; Soto and Sonnenschein 2010; Spencer et al. 2011). Endocrine disruption can also have adverse effects on the fetus or newborn because of the delicate balance of hormones required during critical developmental windows (Bigsby et al. 1999; Chandrasekar et al. 2011; Cooper and Kavlock 1997; Mahoney and Padmanabhan 2010). For example, studies have demonstrated that thyroid hormone insufficiency during pregnancy may lead to adverse neurological outcomes in children (Haddow et al. 1999).

The Federal Food, Drug, and Cosmetic Act (FFDCA 1996), as amended by the Food Quality Protection Act (FQPA 1996), and the Safe Drinking Water Act Amendments (SDWA 1996), requires the U.S. Environmental Protection Agency (EPA) to determine whether certain substances may have an effect in humans similar to that produced by a naturally occurring estrogen, or other such endocrine effects (FFDCA 1996). In response, the U.S. EPA formed the Endocrine Disruptor Screening Program (EDSP) (U.S. EPA 2012b). The EDSP is a two-tiered program that requires chemical manufacturers to submit or generate data on a suite of both *in vivo* and *in vitro* assays. The first phase of EDSP assays are designated as the Tier 1 screening (T1S) battery (U.S. EPA 2012c). These tests identify chemicals with the potential to interact with endocrine pathways or mechanisms, and focus on disruption of estrogen, androgen, and thyroid hormone pathways. Based on a weight-of-evidence approach, chemicals showing positive activity in T1S assays could then be subject to more complex Tier 2 tests (U.S. EPA 2011b). The European Commission is continuing the implementation of the European Union’s Community Strategy for Endocrine Disrupters, which includes the establishment of a priority list of substances for further evaluation and assay development and validation (European Commission 2012). In addition, the European Commission is working toward defining specific criteria to identify endocrine disruptors within a legislative framework, drawing on current scientific opinion (Kortenkamp et al. 2011).

The U.S. EPA estimates that the statutory requirements and discretionary authorities through passage of the FQPA and its amendments and the SDWA will require the EDSP to screen as many as 9,700 environmental chemicals. Generating the data required under the current testing guidelines will be expensive and time-consuming, and it will require significant animal resources (U.S. EPA 2011a). To date, chemicals have been nominated by the U.S. EPA for EDSP T1S on the basis of exposure potential or registration status. Because of fiscal and time constraints, the U.S. EPA is considering using endocrine-related *in vitro* high throughput screening (HTS) assays and *in silico* models to prioritize chemicals for testing in T1S (U.S. EPA 2011a). There has been a significant improvement in HTS technologies since the U.S. EPA began work on developing and implementing the EDSP. In 2007, the National Research Council Report *Toxicity Testing in the 21st Century: A Vision and a Strategy* (National Research Council 2007) acknowledged these advances and recommended that the agency develop a strategy to use modern molecular-based screening methods to reduce, and ultimately replace, the reliance on whole-animal toxicity testing. The U.S. EPA’s ToxCast program (U.S. EPA 2012e), and the U.S. government’s cross-agency Tox21 program (U.S. EPA 2012d) are using HTS assays and developing computational tools to predict chemical hazard, to characterize a diverse set of toxicity pathways, and to prioritize the toxicity testing of environmental chemicals (Huang et al. 2011; U.S. EPA 2012d). Included in these programs are assays that cover toxicity pathways involving estrogen, androgen, and thyroid hormone receptors, as well as targets within the steroidogenesis pathway. The current ToxCast chemical library covers approximately 17% of the chemicals subject to the EDSP, and the larger Tox21 chemical library covers approximately 53% of the chemicals subject to EDSP. Assay technologies include competitive binding, reporter gene, and enzyme inhibition assays. The comparison of HTS assays, endocrine-related modes of action (MOA) and EDSP T1S is shown in Figure 1. An endocrine MOA consists of a series of molecular initiating events relevant for estrogen, androgen, thyroid, or steroidogenic pathways. These assays do not represent their respective MOA in its entirety, but are used to detect chemicals capable of perturbing a particular MOA. In the present study, we investigated the predictive ability of ToxCast HTS assays for end points tested in EDSP T1S, and we tested the hypothesis that if a chemical activates the estrogen or androgen receptor *in vitro,* estrogen- and androgen-related effects will occur in *in vivo* bioassays. Ideally, HTS tests should be highly reproducible and yield a minimal number of false-positive (specificity) and false-negative (sensitivity) chemicals.

**Figure 1:**
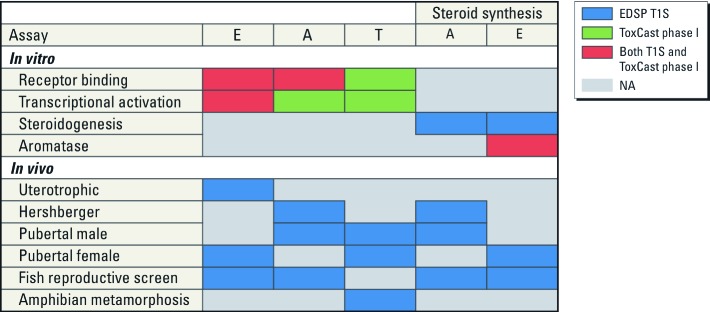
Overlap between EDSP T1S assays and ToxCast phase I assays by endocrine MOAs. Abbreviations: A, androgen; E, estrogen; NA, not applicable; T, thyroid. Colors indicate the type of endocrine MOA data.

Previous studies have suggested the use of HTS assays for identifying endocrine disrupting potential. For example, the ReProTect project developed within the 6th European Framework Program tested 14 *in vitro* assays using 10 prototype compounds to determine feasibility for a reproductive screening program (Schenk et al. 2010). Those *in vitro* assays were grouped into three segments of the reproductive cycle: endocrine disruption, fertility, and embryonic development. The results of ReProTect showed, at least for the 10 prototype chemicals, that appropriate *in vitro* assay selection can effectively group compounds based on known reproductive toxicity (Schenk et al. 2010).

HTS assays are useful for identifying chemical impacts on molecular initiating events in biological or toxicological pathways. Combinations of HTS assays measuring competitive ligand binding, reporter gene activation, and enzyme inhibition can be used to characterize chemical potential for endocrine disruption. These chemical characterizations can then be quantitatively evaluated by investigating associations with guideline EDSP T1S assay results. The aim of the present study was to use this data-driven approach to identify candidate MOAs for predictive modeling efforts, which subsequently will be used to prioritize chemicals for further endocrine-related testing.

## Methods

*Chemical selection.* In this study we used data from the ToxCast Phase I chemical library, containing data for 309 unique chemical structures (U.S. EPA 2012f). Most of these chemicals are either current or former active ingredients in food-use pesticides that were designed to be bioactive, or they are industrial chemicals that are environmentally relevant. Details of the chemical library were reported by Judson et al. (2009). Data on an additional 23 reference chemicals were included that were tested in a separate study (Judson et al. 2010), 17 of which were not in the ToxCast Phase I library. CAS registry numbers (CASRN) for the ToxCast Phase 1 chemicals and the additional 17 chemicals are available online in Supplemental_File_1.csv (Rotroff et al. 2012).

*Guideline and non-guideline endocrine assays.* Data from guideline endocrine-related *in vitro* and *in vivo* studies were extracted from EDSP Tier 1 validation reports from the U.S. EPA EDSP web site (U.S. EPA 2012a). Non-guideline studies were obtained from open literature by querying PubMed (http://www.ncbi.nlm.nih.gov/pubmed) and Google Scholar (http://scholar.google.com/) using the following terms: (any chemical name or CASRN in the 309) AND (“*in vitro”* OR “*in vivo”*) AND (“estrogen” OR “androgen” OR “uterotrophic” OR “Hershberger” OR “steroidogenesis” OR “thyroid hormone”). The automated search found a wide variety of studies representing 2,113 individual studies. The list of studies was manually curated to remove studies that did not contain data usable for the current analysis, leaving 248 unique studies (e.g., studies of mixtures without testing compounds individually, studies that mentioned the chemical but did not test it in a bioassay, studies measuring bioaccumulation). Studies that identified their methods as following the Organisation for Economic Co-operation and Development (OECD) guidelines (Kanno et al. 2001, 2003; OECD 1999, 2001, 2003, 2007) or EDSP protocols were grouped together with EDSP T1S data for the guideline analysis. When available, PubMed identifiers (PMID) were used as unique annotations for each report. For the few instances when no PMID was available or for each EDSP T1S validation report, a unique identifying number was generated. The citation information for all documents used in the analysis is available online in Supplemental_File_2.txt (Rotroff et al. 2012).

Guideline endocrine-related assays gathered from EDSP validation reports and OECD guideline studies were categorized according to whether they tested estrogen-, androgen-, steroidogenesis-, or thyroid-related MOAs (guideline-E, guideline-A, guideline-S, guideline-T, respectively). Additional information captured included study type (e.g., amphibian metamorphosis, reporter gene), assay type (e.g., serum levels, organ weight), species, strain, cell type, target, and whether or not it was an EDSP/OECD guideline study. Chemical potency [e.g., concentration at half-maximum activity (AC_50_), lowest effective concentration] for a given end point was captured as it was represented in the study report along with the maximum concentration/dose tested. In addition, agonist or antagonist responses were noted when applicable. Data from guideline and non-guideline studies were dichotomized as either active if a response was observed, or inactive if no response was observed. If a study investigated multiple end points for a given endocrine MOA and produced at least one statistically significant end point, then that study–chemical–MOA combination was considered active. Activity/inactivity was determined based on the presence of a statistically significant response or was based on the study author’s conclusion. Data were further annotated as having a hit value of either 1 or 0 for active and inactive, respectively. We combined all guideline and non-guideline literature studies to have a single hit value for each study–chemical–MOA combination. Data that were conflicting or otherwise unclear were included in the data table but annotated as such, and removed from analyses. The data obtained from guideline endocrine-related studies and other non-guideline literature reports are available online in Supplemental_File_3.csv (Rotroff et al. 2012).

*ToxCast* in vitro *assays.* HTS competitive binding, enzyme inhibition, and reporter gene assays representing estrogen-, androgen-, steroidogenesis-, or thyroid-related end points (HTS-E, HTS-A, HTS-S, HTS-T, respectively) were selected as a subset of the > 500 HTS assays generated by the ToxCast program (ToxCastDB v.17; U.S. EPA 2012e) [see Supplemental_File_1.csv (Rotroff et al. 2012)]. The details and a description of each assay are reported in Table 1.

**Table 1:** Summary of endocrine-related HTS assays.

					Chemicals tested (n)		
ToxCast assay	Assigned MOA	Species	Assay target	Assay technology	Unique	Overlapping with EDSP/OECD	Overlapping with active chemicals in ToxCast
ATG_AR_TRANS	HTS‑A	Human	Androgen receptor-agonist	Multiplexed reporter gene assay	309a	13	0
NCGC_AR_Agonist	HTS-A	Human	Androgen receptor-agonist	GAL4 BLAM reporter gene assay	309	13	0
NCGC_AR_Antagonist	HTS-A	Human	Androgen receptor-antagonist	GAL4 BLAM reporter gene assay	309	13	5
NVS_NR_hAR	HTS-A	Human	Androgen receptor	Competitive binding	309	13	6
NVS_NR_rAR	HTS-A	Rat	Androgen receptor	Competitive binding	309	13	1
ATG_ERa_TRANS	HTS-E	Human	Estrogen receptor-α	Multiplexed reporter gene assay	326b	21	12
ATG_ERE_CIS	HTS-E	Human	Estrogen receptor response element	Multiplexed reporter gene assay	326b	21	11
ATG_ERRa_TRANS	HTS-E	Human	Estrogen related receptor-α	Multiplexed reporter gene assay	326b	21	0
ATG_ERRg_TRANS	HTS-E	Human	Estrogen related receptor-γ	Multiplexed reporter gene assay	326b	21	0
NCGC_ERalpha_Agonist	HTS-E	Human	Estrogen receptor-α-agonist	GAL4 BLAM reporter gene assay	326b	21	7
NCGC_ERalpha_Antagonist	HTS-E	Human	Estrogen receptor-α-antagonist	GAL4 BLAM reporter gene assay	309	15	4
NVS_NR_bER	HTS-E	Bovine	Estrogen receptor	Competitive binding	316b	17	1
NVS_NR_hER	HTS-E	Human	Estrogen receptor	Competitive binding	326b	21	4
NVS_NR_mERa	HTS-E	Mouse	Estrogen receptor-α	Competitive binding	316b	17	1
NVS_ADME_hCYP19A1	HTS-S	Human	Aromatase	Enzyme Inhibition	309	17	1
NCGC_TRbeta_Agonist	HTS-T	Human	Thyroid hormone receptor-β-agonist	GAL4 BLAM reporter gene assay	309	8	0
NCGC_TRbeta_Antagonist	HTS-T	Human	Thyroid hormone receptor-β-antagonist	GAL4 BLAM reporter gene assay	309	8	0
NVS_NR_hTRa	HTS-T	Human	Thyroid hormone receptor-α-antagonist	Receptor activation	309	8	0
aAdditional reference compounds from Judson et al. (2010) were run but not included because this is the only androgen-related HTS assay that tested these chemicals. bIncludes additional reference compounds from Judson et al. (2010).											

For chemicals that produced a statistically significant and concentration-dependent response in a given assay, the AC_50_ was recorded. The criteria for determining the activity of a compound are assay platform dependent [see Supplemental Material, Appendix A, for further details (http://dx.doi.org/10.1289/ehp.1205065)]. The data were then dichotomized so that if an AC_50_ was present for a given chemical end point concentration, a 1 was reported; if no response was observed, a 0 was reported. Chemicals tested in triplicate for quality control purposes were designated 1 or 0 on a majority basis. Chemicals that were run in duplicate with at least one sample producing an AC_50_ were designated as a 1. Experimental methods for each assay used are provided in Supplemental Material, Appendix A (http://dx.doi.org/10.1289/ehp.1205065).

**Figure 2:**
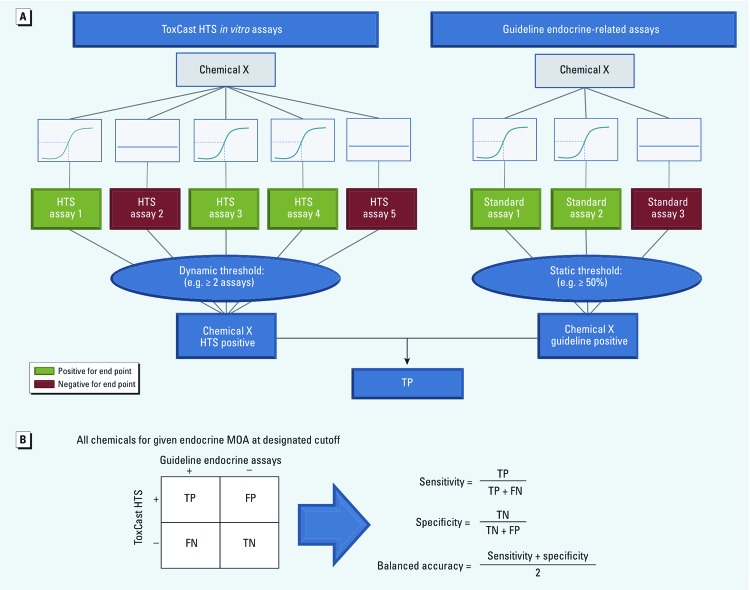
Illustration of the balanced optimization model used to analyze predictive capacity of endocrine-related ToxCast assays. Multiple assays and study reports were available for each chemical–MOA combination. (*A*) Snapshot of a step in this modeling/optimization process, in which chemical X is positive in three of five HTS assays and two of three guideline reports. In this example, the dynamic HTS threshold is at least two positive assays and the guideline threshold is at least 50% positive reports, so chemical X is considered a true positive (TP). With less than two positive assays, chemical X would be a false negative (FN); < 50% positive reports would produce a false positive (FP); and if both were negative according to this criteria, chemical X would be a true negative (TN). (*B*) Method for tabulating results for all chemicals (e.g., chemical X would be counted in the TP portion of the contingency table) to arrive at an estimate of balanced accuracy for each set of threshold parameters.

*Model development.* We performed an iterative, balanced optimization analysis to determine the ability of ToxCast HTS assays to correctly classify the results of guideline endocrine-related assays while maintaining balance between sensitivity and specificity. The process for this analysis is illustrated in Figure 2. Because each HTS endocrine MOA may have multiple ToxCast HTS assays, we used disjunctive logic employing varied weight-of-evidence thresholds to determine optimal predictive performance. This model tested variable thresholds for the HTS ToxCast assay results represented as unweighted binary data, while the guideline or non-guideline endocrine-related assay results remained static. Initially, the model began with a threshold criterion of one positive ToxCast HTS assay out of the total number of ToxCast HTS assays for a chemical to be considered to perturb a given MOA. Once calculated, the model was then re-run with increasing increments of one assay until all ToxCast HTS assays for a given endocrine MOA were required to be positive for a chemical to be considered to perturb the given MOA. As the threshold for a positive call was increased, a larger weight of evidence was required for a chemical to be considered a “hit” for perturbing the given endocrine MOA. An exception was made for guideline pubertal studies and the ToxCast NVS_NR_hAR assay. Guideline pubertal studies test for effects that can arise through multiple different endocrine-related pathways. For this reason, if a chemical was considered positive in the pubertal assay and the result conflicted with other guideline studies (e.g., receptor binding, reporter gene), the pubertal assay was not included in the weight of evidence. The ToxCast NVS_NR_hAR assay is a human androgen receptor binding assay in the LNCaP prostatic cell line. The androgen receptor in this cell line is known to bind to steroid hormones other than androgens (Veldscholte et al. 1992). For this reason, if a compound was negative in all other HTS-A assays, the result for the NVS_NR_hAR assay was not included in the weight-of-evidence.

For a specific set of criteria across all overlapping chemicals, we calculated sensitivity, specificity, and balanced accuracy (BA) as measures of model performance (Figure 2B). The guideline analysis was performed comparing ToxCast HTS assays and guideline endocrine assays gathered from EDSP validation reports and OECD guideline studies. We also conducted a separate non-guideline analysis comparing ToxCast HTS assays with assays from non-guideline studies. Many of the EDSP/OECD guideline studies and those reported in non-guideline literature used multiple studies/assays for each chemical–MOA combination. Because separate studies are not always in agreement relative to a chemical–MOA perturbation, the model was run using two scenarios: *a*) Any positive report for a chemical resulted in a positive call for the chemical–MOA combination; or *b*) > 50% (threshold > 0.50) of guideline or non-guideline endocrine-related studies or assays must report the chemical to be active for a given endocrine MOA.

For each threshold criteria the number of true positives (TP), false positives (FP), true negatives (TN), and false negatives (FN) were calculated. A TP was any chemical determined to be positive in the ToxCast HTS assays and was also positive in guideline endocrine reports. An FP was positive in ToxCast but reported as negative in the guideline endocrine reports. If a chemical was determined to be negative in the ToxCast HTS assays and positive in the guideline endocrine reports, it was recorded as an FN. Last, a TN was any chemical negative in the ToxCast HTS assays and negative in the guideline endocrine reports. At each threshold combination, all of the available chemicals were classified as TP, FP, TN, or FN and were used to calculate sensitivity, specificity, and BA as a measure of model performance.

**Figure 3:**
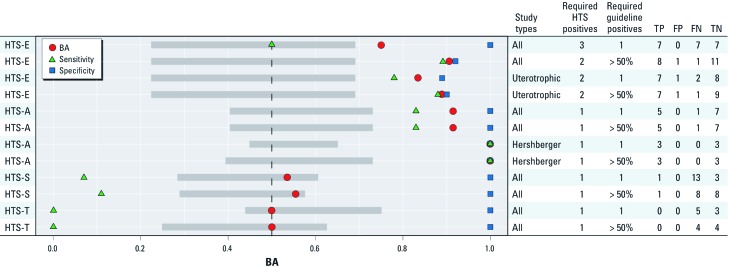
Forest plot illustrating the performance—as measured by sensitivity, specificity, and BA—of ToxCast endocrine-related assays for predicting outcomes captured in EDSP/OECD guideline studies. Symbols represent the optimal BA obtained across all threshold combinations and the corresponding sensitivity and specificity at the same threshold. Gray boxes indicate 95% confidence intervals around permuted BA distributions. Analyses designated “All” include all available assays for the stated endocrine MOA. A value of > 50% “required guideline positives” indicates that > 50% of the studies had to report a positive result for a chemical to be considered a positive in the analysis. If the “required guideline positives” value is 1, any study reporting a positive resulted in the chemical being considered positive in the analysis. A separate analysis compared only uterotrophic and Hershberger analyses (right). The tests listed on the left represent replicate MOA with test conditions annotated under “Required HTS Positives” and “Required guideline positives.”

**Figure 4:**
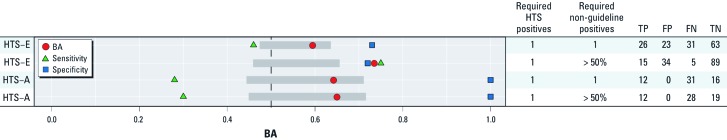
Forest plot illustrating the performance—as measured by sensitivity, specificity, and BA—of ToxCast endocrine-related assays for predicting outcomes captured in non-guideline endocrine studies. Symbols represent the optimal BA obtained across all threshold combinations and the corresponding sensitivity and specificity at the same threshold. Gray boxes indicate 95% confidence intervals around permuted BA distributions. A value of > 50% “required non-guideline positives” indicates that > 50% of the studies had to report a positive result for a chemical to be considered a positive in the analysis. If the “required non-guideline positives” value is 1, any study reporting a positive resulted in the chemical being considered positive in the analysis. The tests listed on the left represent replicate MOA with test conditions annotated under “Required HTS Positives” and “Required non-guideline positives.”

*Statistical analysis.* To identify statistically significant BA values, we performed a permutation test. The test randomized which ToxCast assays were associated with guideline endocrine studies or biomedical literature for each endocrine MOA in order to determine whether or not a randomly chosen set of assays from the > 500 ToxCast end points would likely produce a similar association. The BA calculation based on random assay associations was performed using the same number of ToxCast assays as the model and with the same threshold criteria. Assays were permuted 10,000 times to build the random BA population distribution, and the percentile where the model BA fell among this distribution was calculated to provide a *p*-value. A *p*-value of < 0.01 was considered statistically significant. The distributions developed from the permutation tests were used to define the confidence intervals in Figures 3 and 4.

## Results

*Data collection.* Data covering guideline endocrine-related *in vitro* and *in vivo* assays was extracted from documents used in EDSP Tier 1 validation or conducted according to OECD guidelines. We found a total of 40 studies covering 154 unique chemicals, resulting in a total of 1,246 captured end points. Table 2 shows the chemical overlap between the ToxCast chemical library and the chemicals captured from guideline and non-guideline studies. Twenty-one chemicals available from EDSP validation documents and other OECD guideline studies covering the guideline-E MOA overlapped with the ToxCast HTS-E assays. Thirteen chemicals overlapped in the corresponding guideline-A assays, 8 in the guideline-T assays, and 17 in the guideline-S assays. We extracted additional data used in a separate analysis from a total of 215 non-guideline studies [see Supplemental_File_3.csv (Rotroff et al. 2012)].

**Table 2:** Summary of the endocrine literature survey.

Endocrine modes of action	No. of documents	No. of data points	No. of unique chemicals from literature survey	No. of chemicals overlapping with ToxCast
Estrogenicity	18 (108)	410 (979)	104 (158)	21 (143)
Androgenicity	22 (54)	571 (301)	60 (73)	13 (59)
Steroidogenesis	10 (32)	123 (251)	44 (61)	17 (55)
Thyroid	7 (48)	142 (190)	27 (57)	8 (47)
All	40 (215)	1,246 (1,721)	154 (182)	35 (157)
Values represent guideline (non-guideline) studies.								

*Model results.* The results presented in Figure 3 demonstrate the predictive ability of ToxCast HTS-E and HTS-A assays relative to the corresponding endocrine MOA in the guideline endocrine-related studies. Detailed results from the univariate model with guideline studies are available online in Supplemental_File_4.csv (Rotroff et al. 2012).

*Comparison of HTS and guideline endocrine assays.* For HTS-E end points, we obtained an optimal BA of 0.91 (*p* < 0.001) with a sensitivity of 0.89 and a specificity of 0.92, a threshold of two positives for ToxCast HTS-E assays, and > 50% for guideline-E studies (Figure 3). This means a minimum of two ToxCast HTS-E assays must report an AC_50_ value for a chemical to be considered positive, and > 50% of guideline-E assays must be reported as positive in the EDSP validation reports or OECD guideline studies. Overlapping HTS-E and HTS-A chemicals and corresponding performance in the HTS and guideline studies is provided in Supplemental Material, Appendix C and Tables S2 and S3 (http://dx.doi.org/10.1289/ehp.1205065). Twenty-one guideline-E–related chemicals overlapped with ToxCast Phase I chemicals. One chemical, chlorpyrifos-methyl (CASRN 5598-13-0), was misclassified as a positive (FP) and one chemical, prochloraz (CASRN 67747-09-5), was misclassified as a negative (FN) by this set of ToxCast assays. If the goal was to optimize sensitivity, a threshold criteria of one ToxCast HTS-E assay and > 50% of guideline-E would produce a perfect sensitivity of 1, but specificity drops to 0.5 across this set of ToxCast HTS-E assays [see Supplemental_File_4.csv (Rotroff et al. 2012)]. An additional analysis was conducted in which the threshold criteria for the guideline-E assays lowered from > 50% to any single positive report resulted in a positive call. This lowers the sensitivity from 0.89 to 0.5, and the overall BA drops to 0.75 (Figure 3).

Figure 3 demonstrates the predictive ability of the ToxCast HTS-A assays and the guideline-A results. The optimal predictive ability of the ToxCast HTS-A assays was reached with a threshold of one HTS-A assay and a threshold > 50% for the guideline-A assays. This set of criteria produced a BA of 0.92 (*p* < 0.001), with a sensitivity of 0.83 and specificity of 1 (See Supplemental Material, Appendix C, Table S3) (http://dx.doi.org/10.1289/ehp.1205065). The results for HTS-S and HTS-T were not statistically significant among any of the analyses, with BAs of 0.56 (*p* > 0.01) and 0.50 (*p* > 0.01), respectively [see Supplemental_File_4.csv (Rotroff et al. 2012)].

*Comparison of HTS and uterotrophic and Hershberger assays.* A separate analysis was conducted to determine the predictive capability of the ToxCast HTS-E assays to detect positive and negative chemicals reported in EDSP/OECD guideline uterotrophic assays (Figure 3). Eighteen chemicals were available for comparison, and the optimal thresholds for HTS-E produced a BA of 0.9 (*p* < 0.001), with a sensitivity and a specificity of 0.88 and 0.9, respectively.

In addition, we determined the predictive ability of ToxCast HTS-A assays for EDSP/OECD guideline Hershberger results. Although, only six chemicals were available for comparison, the analysis resulted in a BA of 1 (*p* < 0.001), with a perfect measure of sensitivity and specificity with thresholds of one positive assay required for both HTS-A and EDSP/OECD guideline Hershberger reports (Figure 3).

*Comparison of HTS and non-guideline assays.* Predictive modeling results for non-guideline studies in the biomedical literature are presented in Figure 4. All results from the analysis with non-guideline studies are available online in Supplemental_File_5.csv (Rotroff et al. 2012). The HTS-E MOA produced a maximum BA of 0.74 (*p* < 0.01), with at least one ToxCast assay being positive (ToxCast HTS-E threshold of 1) and a literature threshold of > 50%. These criteria produced a sensitivity of 0.75 and a specificity of 0.72. Because of the wide range of test conditions, assay technologies, and species present in the open-literature, sensitivity was lower than in the guideline studies. This is apparent because of the model optimization that occurred with only one HTS-E assay required for a positive classification, compared with optimizing at two assays in the guideline analysis. We observed an overall concordance of 0.7 between the guideline-E assay results and the estrogen-related literature results given the stated thresholds (data not shown).

The optimal BA reached 0.65 (*p* > 0.01) with the ToxCast HTS-A assays threshold of 1 and and androgen-related literature threshold > 50%. At these thresholds, sensitivity was low (0.3) but specificity was 1 (Figure 4). There was a concordance between chemical classifications for guideline-A reports and non-guideline reports of 0.77 at the reported thresholds of > 50% (data not shown).

## Discussion

The results of this study demonstrate that ToxCast *in vitro* assays perform adequately to prioritize chemicals for further EDSP T1S for estrogen and androgen activity, and these HTS assays are predictive of the likelihood of a positive or negative finding in more resource-intensive assays. Additional HTS assays will be needed to predict steroidogenic and thyroid activity of chemicals. Methods for prioritizing chemicals based on a broad range of ToxCast HTS assays, in combination with physical–chemical properties, have been previously developed (Reif et al. 2010). Other efforts are also under way to develop more sophisticated, pathway-based predictive models that would be more suitable for supporting regulatory decision making. The present study demonstrates the MOA for which these models would be expected to succeed, and for which areas need additional technologies before a sufficient screening tool would be expected to be successful. This information can now be used for more focused follow-up efforts to identify endocrine-related MOAs for prioritization.

The HTS-E and HTS-A assays demonstrate a high degree of association with the guideline-E and guideline-A assays. The two types of misclassifications, FP and FN, are important because they highlight shortcomings in the model or further specify the domain of applicability. FPs are compounds predicted to be active but that were not active in this analysis based on the threshold of EDSP/OECD reports or literature data. These are significant because an FP could lead to unnecessary testing in more resource intensive assays, and FNs are of concern because they represent potentially active chemicals that would have gone undetected.

The HTS-E model correctly classified 90% of chemicals, and only 2 of 21 chemicals were misclassified as FP or FN. Chlorpyrifos-methyl was an FP, meaning that it was predicted to be estrogenic by ToxCast HTS-E assays but was not positive in the only guideline-E report, which was a uterotrophic study by Kang et al. (2004) [see Supplemental Material, Appendix C, Table S2 (http://dx.doi.org/10.1289/ehp.1205065)]. This same chemical was reported to be inactive in all of the extracted non-guideline-E literature data (active in 0 of 4 available assays). Chlorpyrifos-methyl was inactive in all ToxCast HTS-E assays except for the Attagene ERα TRANS and CIS reporter gene assays, which resulted in the subsequent positive call.

Non-guideline estrogen-related literature for prochloraz reported observations of ERα antagonism in some reporter gene and proliferation assays (Bonefeld-Jorgensen et al. 2005; Kjaerstad et al. 2010), but other studies did not observe activity in reporter gene assays (Andersen et al. 2002; Kojima et al. 2004; Lemaire et al. 2006; Petit et al. 1997) or proliferation assays (Andersen et al. 2002; Vinggaard et al. 1999) [see Supplemental_File_3.csv (Rotroff et al. 2012)]. Prochloraz was an FN in this analysis because it was active in the NCGC_ERalpha_Antagonist assay but negative in all other ToxCast HTS-E binding and reporter gene assays [see Supplemental_File_1.csv (Rotroff et al. 2012)]. Prochloraz tested positive in the only guideline-E assay available [see Supplemental Material, Appendix C, Table S2 (http://dx.doi.org/10.1289/ehp.1205065)]. This EDSP/OECD fathead minnow assay showed altered fecundity, vitellogenin, and oocyte atresia after prochloraz treatment (U.S. EPA 2007). Prochloraz is known to disrupt steroidogenesis through inhibition of CYP (cytochrome P450) 17 hydroxylase and aromatase, preventing the critical conversion of progesterone to 17α-hydroxyprogesterone and testosterone to 17β-estradiol, respectively (Blystone et al. 2007; Sanderson et al. 2002). The fathead minnow assay likely detected this non–receptor-mediated mechanism of estrogen disruption, and this mechanism of action would not have been expected to be detected in the current set of ToxCast HTS-E assays. Prochloraz was the only compound misclassified in the HTS-A analysis, and the effects observed in the reproductive study in male fish are likely a result of the same steroidogenic perturbations. Prochloraz was correctly identified by the ToxCast aromatase enzyme inhibition assay, which was grouped with the HTS-S–related MOA.

Although a limited number of chemicals was available for comparison, we found a strong association between the ToxCast HTS-E and HTS-A assays with EDSP/OECD guideline uterotrophic and Hershberger studies. Eighteen chemicals were available for comparison between ToxCast HTS-E and guideline uterotrophic assays and only two were misclassified [see Supplemental Material, Appendix C, Table S2 (http://dx.doi.org/10.1289/ehp.1205065)]. Six chemicals were available for analysis between ToxCast HTS-A assays and Hershberger responses, and all of these chemicals were classified correctly for a perfect BA of 1 (see Supplemental Material, Appendix C, Table S3).

There are several explanations for why a chemical may be misclassified by the ToxCast HTS models. In some scenarios a chemical may not have been tested at concentrations high enough to exhibit a response in ToxCast assays. Inconsistencies could also result from species, tissue, or cell-type differences between the ToxCast and guideline studies. Most of the ToxCast assays use human cell lines or reporter constructs, and some areas of misclassification may result from species differences between these assays and the rodent bioassays. Comparisons of available species between guideline and non-guideline studies are available in Supplemental Material, Appendix B, Table S1 (http://dx.doi.org/10.1289/ehp.1205065). Interspecies differences should be taken into consideration because they may be quite substantial. For example, studies have highlighted not only the importance of tissue and cell distribution and context within an organism for both ER and AR (Kolasa et al. 2003; Zhou et al. 2002) but also the presence of ERα and ERβ splice variants (Saunders et al. 2002). Most *in vitro* assays are limited in their metabolic capabilities, so chemicals that require metabolic activation in order to be active may not be detected. However, methoxychlor and vinclozolin, which become more active with metabolism, were both detected in the HTS-E (see Supplemental Material, Appendix C, Table S2) and HTS-A (see Supplemental Material, Appendix C, Table S3) assays. Furthermore, *in vivo* assays may detect chemicals that perturb endocrine-related end points elicited via toxicity in other organs, such as the liver (Leffert and Alexander 1976; Masuyama et al. 2000; Xie et al. 2003). The assays selected for the present study comprise only a small portion of the overall endocrine pathway domain. Alterations in neuroendocrine or other pathways, as well as some feedback mechanisms, could be affected by a compound and would not be detected by these assays. The methods we used to classify compounds may result in different conclusions than those obtained by the EDSP (U.S. EPA 2011b). Despite these limitations, evidence from the present study indicates that very few chemicals that are active in EDSP T1S go undetected by ToxCast HTS-E and HTS-A assays. Most of the misclassifications appear to be from downstream estrogenic and androgenic effects caused by alterations of upstream steroidogenic enzymes. Most of the active guideline-E and guideline-A chemicals in this data set appear to operate through receptor-mediated pathways and are detectable *in vitro*.

The non-guideline literature analysis demonstrated that ToxCast HTS assays are also predictive of a broader range of endocrine-related assays. As expected, we observed a loss of accuracy in predicting the non-guideline literature analysis compared with the EDSP/OECD guideline studies because the non-guideline literature studies used a wide variety of species, assay protocols, and technologies. An additional factor that led to the loss of sensitivity in the HTS-A non-guideline analysis was the imbalance of positive to negative reports. The guideline study had 6 positives of 13 total chemicals (46%) at > 50% threshold, and the non-guideline reports had 47 positives of 59 total chemicals (80%) at the same threshold. The sensitivity would be expected to improve with a more balanced data set.

This analysis shows that there is a clear need to develop HTS assays capable of detecting steroidogenesis and thyroid disrupting compounds. The current HTS-S related assay within ToxCast is limited to a single cell-free aromatase enzyme activity assay. Aromatase is a key enzyme in the biosynthesis of estrogens from androgens (Schuurmans et al. 1991; Stoker et al. 2000a). However, in addition to aromatase inhibition, other mechanisms of steroidogenesis may be impacted by environmental chemicals that are not tested in our current HTS battery (Stoker et al. 2000a, 2000b). Additional assay technologies that may provide a more comprehensive set of steroidogenesis end points are currently being assessed.

The ToxCast HTS-T assays used in our analysis are composed of thyroid hormone receptor binding and reporter gene assays. A limited number of chemicals was available for comparison between the HTS-T assays and the guideline studies. The inability of the ToxCast HTS-T assay results to associate with compounds thought to disrupt thyroid homeostasis in EDSP/OECD guideline studies suggests that most of these compounds are not acting through thyroid hormone receptor-mediated mechanisms (Paul et al. 2010; Zorrilla et al. 2009). Thyroid hormone homeostasis has been shown to be altered through enhanced or suppressed clearance of thyroid hormone by metabolic enzymes (Saghir et al. 2008; Zorrilla et al. 2009). ToxCast contains HTS assays that measure nuclear receptor activation and metabolic enzyme activity, which could be relevant for thyroid hormone metabolism. However, many chemicals that were active in these *in vitro* ToxCast assays were not associated with adverse outcomes in the *in vivo* literature we reviewed, and the subsequent lack of specificity for thyroid-active chemicals led to their exclusion from this analysis (data not shown).

From these findings, we conclude that most chemicals chosen to validate EDSP T1S assays alter estrogen- and/or androgen-related end points through nuclear receptor-mediated mechanisms and are capable of being efficiently detected by the ToxCast HTS assays. For the purpose of prioritization, it is important to establish sufficient confidence that the assays being utilized are specific and sensitive so that chemicals prioritized for EDSP T1S include those most likely to be active. Although further efforts are needed to improve detection of steroidogenic and thyroid-disrupting chemicals with *in vitro* test systems, our results indicate that ToxCast endocrine assays are highly predictive of chemicals with estrogenic and androgenic receptor-based endocrine MOAs, and that their use in predictive models for endocrine testing would allow efficient prioritizing of chemicals for further testing.

## Supplemental Material

(967 KB) PDF
