# Supplementary material for: Using in Vitro High Throughput Screening Assays to Identify Potential Endocrine-Disrupting Chemicals
Source: Environ Health Perspect. 2012 Sep 28;121(1):7–14. doi: 10.1289/ehp.1205065 (PMC3546348; doi:10.1289/ehp.1205065)
Supplement: (967 KB) PDF [file ehp.1205065.s001.pdf]

Supplemental Material

Using *in Vitro* High-Throughput Screening Assays to Identify Potential Endocrine Disrupting Chemicals

Daniel M. Rotroff [1,2], David J. Dix[2], Keith A. Houck[2], Thomas B. Knudsen[2], Matthew T. Martin[2], Keith W. McLaurin[2], David M. Reif[2], Kevin M. Crofton[3], Amar V. Singh[4], Menghang Xia[5], Ruili Huang[5], Richard S. Judson[2,¶]

[1]Department of Environmental Sciences and Engineering, University of North Carolina, Chapel Hill, North Carolina, USA

[2]United States Environmental Protection Agency, Office of Research and Development, National Center for Computational Toxicology, Research Triangle Park, North Carolina, USA

[3]United States Environmental Protection Agency, Office of Research and Development, National Health and Environmental Effects Research Laboratory, Research Triangle Park, North Carolina, USA

[4]Lockheed Martin, Research Triangle Park, North Carolina, USA

[5]National Center for Advancing Translational Sciences, National Institutes of Health, Bethesda, Maryland, USA

Key Words: Androgen, Endocrine, Estrogen, High-Throughput, In Vitro, ToxCast

Short Title: High-Throughput Endocrine Disruptor Screening

Disclaimer: The views expressed in this article are those of the authors and do not necessarily reflect the views of policies of the U.S. Environmental Protection Agency. Mention of trade names or commercial products does not constitute endorsement or recommendation for use.

¶Corresponding Author
Richard S. Judson, Ph.D.
National Center for Computational Toxicology
Office of Research and Development

U.S. Environmental Protection Agency
109 T.W. Alexander Drive (B205-01)
Research Triangle Park, NC 27711
Phone: (919) 541-3085
Fax: (919) 541-3513
Email: judson.richard@epa.gov

Contents

Appendix A – Supplemental Methods for ToxCast *In Vitro* Assays 3
Appendix A.1: Competitive Binding and Enzyme Inhibition Assays 3
Appendix A.2: Cis- and Trans-activation Reporter Gene Assays 4
Appendix A.2a: Cis- and Trans-activation Hit Filtering 4
Appendix A.3: Agonist and Antagonist Quantitative HTS Reporter Gene Assays 6
Appendix B: Non-Guideline and Guideline Literature Survey 7
Supplemental Material, Table S1: Species Comparisons Between Non-Guideline and Guideline Studies 8
Appendix C: Individual Assay and Chemical Results 8
Appendix C.1: Chemical Results from Overlapping HTS-E and Guideline Reports 8
Supplemental Material, Table S2: Chemical Results Overlapping HTS-E and Guideline-E Studies 9
Appendix C.2: Chemical Results from Overlapping HTS-A and Guideline Reports 10
Supplemental Material, Table S3: Chemical Results Overlapping HTS-A and Guideline-A Studies ... 11
Appendix D: Links to Additional Supplemental Data Files 12
Upon downloading the .ZIP file, the 5 supplemental data files will be decompressed: 12
Supplemental_File_1.csv: File Containing ToxCast Data Used for Analysis 12
Supplemental_File_2.txt: Citations for Guideline and Non-Guideline Studies 12
Supplemental_File_3.csv: File Containing Guideline and Non-Guideline Data Used for Analysis 12
Supplemental_File_4.csv: Guideline Model Results 12
Supplemental_File_5.csv: Non-Guideline Model Results 12
References 13

Appendix A – Supplemental Methods for ToxCast *In Vitro* Assays

Appendix A.1: **Competitive Binding and Enzyme Inhibition Assays**

HTS competitive binding assays for human, bovine, and mouse estrogen receptor (hER, bER, mERa, respectively), rodent and human androgen receptor (rAR and hAR, respectively), human thyroid hormone receptor-alpha (hTRa), and a human aromatase (hCYP19A1) enzyme inhibition assay (as part of a larger collection of 239 targets) were developed and run by Caliper Discovery Alliances and Services (Hanover, MD). A more complete description of the large set of HTS assays from which these are taken is provided in Knudsen et al. (Knudsen et al. 2011). The hER, bER, mERa receptor binding assays (Catalog Nos. 100-0127, 100-0126, 100-0897) were conducted on extracts of human breast cancer cells, bovine, and mouse uterine membranes, respectively. The ER radioligand assays measure displacement of [^{3}H]-estradiol at final ligand concentrations of 0.1 nM (hER) and 0.7 nM (bER) with the positive reference 17β-estradiol. The hAR and rAR receptor binding assays (Catalog Nos. 100-0167,100-0904) were conducted using human prostate cells (LnCAP) which contain a mutated androgen receptor and recombinant truncated rat receptor, respectively. The AR radioligand assays measure displacement of [^{3}H]-methyltrienolone at final ligand concentrations of 0.3 nM and 4.59 nM, respectively. The TR radioligand assays measure displacement of T3 at final ligand concentration of 15 nM. Reactions were carried out in 10 mM TRIS-HCl (pH 7.4 containing 1.5 mM EDTA, 1.0 mM dithiothreitol and 25 mM sodium molybdate at 0-4 ºC for 18 hr. The reaction is terminated with dextran-coated charcoal and incubated for 20 min at 0-4 ºC to adsorb unbound radioactivity. After centrifugation, the radioactivity remaining bound in the supernatant fraction is determined and compared to reference control values in order to ascertain any interactions of test compound with the ligand-binding site. The hTRa receptor activation assay (Catalog No. 100-0871) conducted using human recombinant thyroid hormone receptor. The hCYP19A1 enzyme inhibition assay (Catalog No. 400-0905) was conducted using recombinant human CYP19A1 enzyme with a substrate (Di(benzyloxymethoxy)fluorescein) concentration of 2.5 µM.

The competitive binding and enzyme inhibition assays were initially run in duplicate at a single concentration (10_M for CYP19A1 assay and 25_M for all others). Assay-chemical combinations meeting a pre-defined threshold of 30%, from the vehicle (DMSO) control signal or if the Z score was at least 2.0 median absolute deviations from the median (30% inhibition or MAD2) were then run in a follow-up screen in singleton concentration–response format with maximum concentration of 20_M for CYP19a1 and 50_M for all others (Knudsen et al. 2011).

Concentration response curves in the follow-up screen constrained the upper and lower asymptotes of the curve between 0- and 20% activity and between 100- and 120% activity, respectively, to allow for consistent extrapolation of the AC50 across assay-chemical combinations. Extrapolated AC50s above the highest concentration tested were allowed if the Emax was greater than 25% activity. Emax is defined in this analysis as the maximal tested response minus the lower asymptote. In order for a response to

report an AC50 and be established as a hit, an Emax of 25% and an R-squared filter of 0.5 must be obtained (Knudsen et al. 2011).

Appendix A.2: **Cis- and Trans-activation Reporter Gene Assays**

Data on a large collection of transcription factor assays, including two ER assays (ERa_TRANS, ERE_CIS), two estrogen related receptor assays (ERRa_TRANS, ERRg_TRANS), and an androgen receptor agonist assay (AR_TRANS) were run on the samples. This collection of a multiplexed reporter gene assays and data on 309 environmental chemicals are described in Martin et al. (Martin et al. 2010). Attagene Inc. (RTP, NC), under contract to the U.S. EPA (Contract Number EP-W-07-049), provided multiplexed reporter transcription unit (RTU) assays (Factorial, patents pending) consisting of 48 human transcription factor DNA binding sites transiently transfected into the HepG2 human liver hepatoma cell line (Romanov et al. 2008). In addition to the Cis-acting reporter genes (CIS), a modification of the approach was used to generate a trans-system (TRANS) with a mammalian one-hybrid assay consisting of an additional 25 RTU library reporting the activity of nuclear receptor (NR) superfamily members (Martin et al. 2010).The human ligand-binding domain of each nuclear receptor was expressed as a chimera with the yeast GAL4 DNA-binding domain that activated in trans a 5XUAS-TATA promoter, which regulated the transcription of a reporter sequence unique to each NR RTU. To ensure the specificity of detection, each individual trans-RTU system including both receptor and reporter gene was separately transfected into suspended cells followed by pooling and plating of the transfected cells prior to screening. A major difference between the CIS and TRANS system is that in CIS activities of endogenous transcription factors are measured, whereas the TRANS assay evaluates changes in activities of exogenous, chimeric NR-Gal4 proteins. Since the HepG2 cell line does not express some nuclear receptors, the CIS assay cannot be used to evaluate these targets, including the androgen receptor. A cytotoxicity assessment was performed at the higher concentrations, qualitatively, to remove confounding data from the downstream analysis process. Additional details on how the cytotoxicity assessment was performed are provided below.

Appendix A.2a: Cis- and Trans-activation Hit Filtering

Two of the ER assays used in our case study are provided by Attagene as part of a multiplexed reporter gene assay(Martin et al. 2010). Attagene provide two assays (labeled TRANS and the CIS) which employ ligand-binding domain and full-length nuclear receptors respectively. The Attagene assays hit an unexpectedly large number of chemicals for certain genes, including ER. For many chemicals, as the concentration approaches the level of cytotoxicity, there is a significant amount of non-specific activity observed. Here we describe our method to use this information to develop a confidence score to filter out non-specific hits. We also make use of 3 assays associated with generalized cell stress that provide additional information that can be used to filter out spurious hits. The confidence filtering process has the following steps:

1. For each chemical, create a histogram of assay hits. For many chemicals, there is a burst of activity at high concentration (typically in the range of 10-100 μM). Create a confidence function which is represented as a Hill curve with 50% point being 0.25 log units lower in concentration

than the peak of the high-concentration activity burst, and with a Hill-slope of 3. The top of the curve is 1 and the bottom depends on the total number of hits (=*n*) for the chemical. For n≤5, bottom is 1 and for n>10, bottom is 0. The bottom is a linear function of *n* for *n*=[6…9]. This function is called $Conf_{Hit}$(chemical, concentration).

2. Create a second confidence function based on the cell stress assays (genes: NFE2EL2, MTF2, CREB3; assay codes: ATG_NRF2_ARE_CIS, ATG_MRE_CIS and ATG_CRE_CIS). If there are 2 or 3 of these active, create a Hill function with midpoint=0.25 log units lower in concentration than the highest concentration of the stress assays and with a Hill-slope of 3. The top is set to 1 and the bottom to 0.5 if 2 cell-stress assays are hit, or to 0 if 3 cell-stress assays are hit. This function is called $Conf_{Stress}$(chemical, concentration).

3. The overall confidence value $Conf_{Overall}$ for each assay is then the product $Conf_{Hit}$(chemical, AC50) x $Conf_{Stress}$(chemical, AC50), where the AC50 for that assay is used.

4. A chemical-assay hit is accepted if the overall confidence value $Conf_{Overall}$ >0.5.

5. For genes such as ER for which there are 2 complementary assays (one CIS and one TRANS), we can further require that both assays are hits.

Out of 339 chemicals tested to date in these assays, 127 were positive in one of the Attagene ER before filtering (105 in ATG_ERa_TRANS and 53 in ATG_ERE_CIS). After filtering, there are 75 total hits (61 in ATG_ERa_TRANS and 36 in ATG_ERE_CIS). There were finally only 20 chemicals that passed the filter for both the CIS and TRANS ER assays. ICCVAM/NICEATM have compiled data from the literature for a list of compounds tested in ER transactivation assays(NICEATM/ICCVAM 2011). The following numbers in parentheses are (positive repots in the literature / total reports in the literature). According to ICCVAM/NICEATM, a chemical is listed as "Positive" if there are 5 or more reports and at least half are positive. It is denoted "Presumed Positive" if there are fewer than 5 reports, but the majority are positive. It is denoted as "Negative" otherwise. Of the 20 chemicals with hits in both CIS and TRANS ER after filtering, 9 are listed as Positive by ICCVAM/NICEATM; 1 is a Presumed Positive; 1 (HPTE) is an active metabolite of a positive (Methoxychlor) and 2 are known to break down to a "Positive" (the nonylphenol ethoxylates Igepal and Tergitol break down to nonylphenols).

The remaining 7 positives include Chlorpyrifos-methyl, Fenamiphos, Pendimethalin and Perfluorooctanoic acid, which are active in the Attagene assays only at high concentrations (37 and 42 μM) and inactive in all 4 other assays. There are literature reports for Chlorpyrifos-methyl (3 negative reports: (Vinggaard et al. 1999; Nishihara et al. 2000; Kojima et al. 2004)), Fenamiphos (1 negative report,(Kojima et al. 2004)), Pendimethalin (1 positive report: (Kojima et al. 2004), and 1 negative report: (Nishihara et al. 2000)). No literature reports for Perfluorooctanoic acid in ER assays were found. Dithiopyr was active in the Attagene assays at intermediate concentrations (20 and 5.2 μM) and inactive in all 4 other assays. No ER literature reports were found. Fenhexamid was active in the Attagene assays

at intermediate concentrations (10 and 9.2 μM) and inactive in the binding assays, but was active in the NCGC reporter gene assay (14.5 μM). Flumetralin followed this same pattern (Attagene AC50s: 5.4 and 2.4 μM and NCGC AC50: 4.5 μM). No literature reports for either of these were found. In summary, there is support for 3 of 7 of these compounds being ER active *in vitro*, independent of the Attagene assays.

In summary, we observed that a large number of chemicals show a positive response in one or both of the Attagene ER assays, but that most of these hits can be ascribed to non-specific activity at high concentrations where generalized cell stress and cytotoxicity are evident. By filtering out these non-specific activities, we are left with a set of positives which have significant support from prior literature or from other assays run as part of this study. The specific parameters using in the filtering algorithm can be adjusted to alter the overall sensitivity and specificity of these assays.

Appendix A.3: **Agonist and Antagonist Quantitative HTS Reporter Gene Assays**

GeneBLAzer® AR-UAS-bla GripTiteTM cell line, GeneBLAzer® ERα-UAS-bla GripTiteTM cell line and GeneBLAzer® TRβ-UAS-bla HEK 293T cell line were obtained from Invitrogen (Carlsbad, CA, USA). Each of these lines stably express the ligand-binding domain (LBD) of the specific human nuclear receptor fused to the DNA-binding domain (DBD) of GAL4 and a beta-lactamase reporter gene under the transcriptional control of an upstream activator sequence (UAS). Binding of agonist to the LBD of the GAL4 (DBD)-NR (LBD) fusion protein causes the fusion protein to bind to the UAS, resulting in expression of beta-lactamase. Assays were conducted in both agonist and antagonist mode. In antagonist mode, an AC50 concentration of the reference ligand was included in the assay. Cells were cultured in medium containing 2% charcoal/dextran-treated FBS, 0.1 mM NEAA and 1 mM sodium pyruvate overnight in the flasks before the assay. The assay was performed in clear bottom black Greiner 1536-well plates. Positive controls for ER-alpha, AR, and TR-beta were 17β-estradiol, R1881 and T3, respectively. Library compounds were measured for their ability to either stimulate or inhibit (in the presence of an AC50 of the appropriated agonist) the reporter gene activity. Compounds were screened in a 15-point titration series from 1 nM to 76 μM in 1536-well format and reporter gene activity determined as previously described (Huang et al., 2011). Data were normalized relative to positive controls (20 nM, 100%, for agonist mode and 0.5nM, 0%, for antagonist mode), and DMSO-only wells (basal, 0% for agonist mode and -100% for antagonist mode). Concentration-response titration points for each compound were fitted to the Hill equation yielding concentrations of half-maximal stimulation (EC_{50}), half-maximal inhibition (IC_{50}) and maximal response (% of control) values (Huang et al. 2011). For agonist activity, the maximal response needed to be at least 20% of the maximal response of the positive control to be considered agonist activity.

Appendix B: Non-Guideline and Guideline Literature Survey

There were a wide variety of study types, assay conditions, and species tested in the Non-guideline literature analysis. These study conditions were captured when available and are thought to contribute to the loss of predictive accuracy compared to the more consistent guideline studies. **Supplemental Material, Table S1** shows a comparison of the species tested in the each of the MOA assessed across both guideline and non-guideline reports.

Test Species in Guideline and Non-Guideline *In Vitro* and *In Vivo* Studies

Organism	Non-Guideline-E	Guideline-E	Non-Guideline-A	Guideline-A
Human	✓	✓	✓	
Bovine	✓		✓	
Atlantic Croaker			✓	
Kelp Bass			✓	
Rat	✓		✓	✓
Fathead Minnow	✓	✓	✓	✓
Mouse	✓		✓	
Rat		✓		
Rainbow Trout	✓			
Rabbit	✓			
Chicken	✓			
Green Anole	✓			
Channel Catfish	✓			
Zebrafish	✓			
Xenopus	✓			
Caiman	✓			
Whiptail Lizard	✓			
Largemouth Bass	✓			

Supplemental Material, Table S1: Species Comparisons Between Non-Guideline and Guideline Studies

Appendix C: Individual Assay and Chemical Results

Appendix C.1: Chemical Results from Overlapping HTS-E and Guideline Reports

For HTS-E endpoints, an optimal BA of 0.91 ($P < 0.001$) was obtained with a sensitivity of 0.89 and specificity of 0.92 with a threshold of 2 positives for ToxCast HTS-E assays and >50% for Guideline-E studies (**Supplemental Material, Table S2**). This means a minimum of 2 ToxCast HTS-E assays must report an AC50 value for a chemical to be considered positive; and greater than 50% of Guideline-E assays must be reported as positive in the EDSP validation reports or OECD guideline studies. A table of overlapping HTS-E and HTS-A chemicals and corresponding performance in the HTS and guideline studies is provided in **Supplemental Material, Table S2**. There were 21 Guideline-E related chemicals that overlapped with the ToxCast Phase I chemicals. One chemical, chlorpyrifos-methyl (5598-13-0), was misclassified as a positive (FP) and one chemical, prochloraz (67747-09-5), was misclassified as a negative (FN) by this set of ToxCast assays.

Chemicals Results Overlapping HTS-E and Guideline-E

Chemical Information		Model Parameters and Results								HTS Assays									Guideline				
CASRN	Name	Endocrine Modality	Required Positives From ToxCast	Required Positives From Literature[c]	BA	Sens	Spec	ToxCast[a]	Guideline[a]	ATG_ERa_TRANS[a]	ATG_ERE_CIS[a]	ATG_ERRa_TRANS[a]	ATG_ERRg_TRANS[a]	NCGC_ERalpha_Agonist[a]	NCGC_ERalpha_Antagonist[a]	NVS_NR_bER[a]	NVS_NR_hER[a]	NVS_NR_mERa[a]	E_Binding[b]	E_Fish Repro[b]	E_Pubertal - F[b,c]	E_Reporter Gene[b]	E_Uterotrophic[b]
1912-24-9	Atrazine	E	2	>50%	0.91	0.89	0.92	0	0	0	0	0	0	0	0	0	0	0	0/1	NA	1/0	0/1	1/0
17804-35-2	Benomyl	E	2	>50%	0.91	0.89	0.92	0	0	0	0	0	0	0	0	0	0	0	0/1	NA	NA	0/1	1/1
80-05-7	Bisphenol A	E	2	>50%	0.91	0.89	0.92	1	1	1	1	0	0	1	1	1	1	1	1/0	NA	0/1	NA	8/1
2425-06-1	Captafol	E	2	>50%	0.91	0.89	0.92	0	0	0	0	0	0	0	0	0	0	0	1/0	NA	NA	0/1	0/1
5598-13-0	Chlorpyrifos-methyl	E	2	>50%	0.91	0.89	0.92	1	0	1	1	0	0	0	0	0	0	0	NA	NA	NA	NA	0/1
84-74-2	Dibutyl phthalate	E	2	>50%	0.91	0.89	0.92	0	0	1	0	0	0	0	0	0	0	0	NA	NA	NA	NA	0/3
117-81-7	Diethylhexyl phthalate	E	2	>50%	0.91	0.89	0.92	0	0	0	0	0	0	0	0	0	0	0	1/0	NA	NA	0/1	0/1
66230-04-4	Esfenvalerate	E	2	>50%	0.91	0.89	0.92	0	0	1	0	0	0	0	0	0	0	0	NA	NA	NA	NA	0/1
60168-88-9	Fenarimol	E	2	>50%	0.91	0.89	0.92	1	1	1	1	0	0	1	1	0	0	0	NA	NA	1/0	NA	NA
72-43-5	Methoxychlor	E	2	>50%	0.91	0.89	0.92	1	1	1	1	0	0	1	1	0	0	0	NA	NA	1/0	NA	7/0
40487-42-1	Pendimethalin	E	2	>50%	0.91	0.89	0.92	1	1	1	1	0	0	0	0	0	0	0	NA	NA	NA	NA	1/0
52645-53-1	Permethrin	E	2	>50%	0.91	0.89	0.92	0	0	1	0	0	0	0	0	0	0	0	NA	NA	NA	NA	0/1
67747-09-5	Prochloraz	E	2	>50%	0.91	0.89	0.92	0	1	0	0	0	0	0	1	0	0	0	NA	1/0	NA	NA	NA
3380-34-5	Triclosan	E	2	>50%	0.91	0.89	0.92	0	0	0	0	0	0	0	0	0	0	0	NA	NA	1/0	NA	0/1
50471-44-8	Vinclozolin	E	2	>50%	0.91	0.89	0.92	0	0	0	1	0	0	0	0	0	0	0	NA	0/1	NA	NA	NA
104-40-5	4 Nonylphenol	E	2	>50%	0.91	0.89	0.92	0	0	0	1	0	0	0	NA	0	0	0	1/0	NA	NA	NA	NA
140-66-9	4-(tert-octyl)Phenol	E	2	>50%	0.91	0.89	0.92	1	1	1	1	0	0	1	NA	NA	1	NA	NA	1/0	NA	NA	2/0
521-18-6	5a-androstan-17b-ol-3-one	E	2	>50%	0.91	0.89	0.92	1	1	1	1	0	0	1	NA	NA	0	NA	NA	NA	NA	NA	1/0
50-28-2	b-estradiol	E	2	>50%	0.91	0.89	0.92	1	1	1	1	0	0	1	NA	NA	1	NA	1/0	NA	NA	NA	3/0
13311-84-7	Flutamide	E	2	>50%	0.91	0.89	0.92	0	0	0	0	0	0	0	NA	0	0	0	0/1	NA	NA	0/1	0/1
446-72-0	Genistein	E	2	>50%	0.91	0.89	0.92	1	1	1	1	0	0	1	NA	NA	1	NA	1/0	NA	NA	NA	5/0

[a] 1 indicates positive result, 0 indicates negative result

[b] (# of positive reports/# number of negative reports)

[c] Conflicting pubertal studies were ignored (See methods)

Supplemental Material, Table S2: Chemical Results Overlapping HTS-E and Guideline-E Studies

Appendix C.2: Chemical Results from Overlapping HTS-A and Guideline Reports

The optimal predictive ability of the ToxCast HTS-A assays was reached with a threshold of 1 HTS-A assay and a threshold > 50% for the Guideline-A assays. This set of criteria produced a BA of 0.92 ($P < 0.001$) with a sensitivity of 0.83 and specificity of 1 (**Supplemental Material, Table S3**). Prochloraz was the only compound misclassified in the HTS-A analysis, and the effects observed in the male fish reproductive study are likely a result of the same steroidogenic perturbations. Prochloraz was correctly identified by ToxCast aromatase enzyme inhibition assay which was grouped with the HTS-S related MOA.

Chemicals Results Overlapping HTS-A and Guideline-A																			
Chemical Information		Model Paramters and Results								HTS Assays					Guideline				
CASRN	Name	Endocrine Modality	Required Positives From ToxCast	Required Positives From Literature[c,d]	BA	Sens	Spec	ToxCast[a]	Guideline[a]	ATG_AR_TRANS[a]	NCGC_AR_Agonist[a]	NCGC_AR_Antagonist[a]	NVS_NR_hAR[a,d]	NVS_NR_rAR[a]	A_15-day adult intact male[b]	A_Binding[b]	A_Fish Repro[b]	A_Hershberger[b]	A_Pubertal-M[b,c]
1912-24-9	Atrazine	A	1	>50%	0.92	0.83	1	0	0	0	0	0	0	0	NA	0/1	NA	NA	2/0
80-05-7	Bisphenol A	A	1	>50%	0.92	0.83	1	1	1	0	0	1	1	0	NA	1/0	NA	NA	NA
84-74-2	Dibutyl phthalate	A	1	>50%	0.92	0.83	1	0	0	0	0	0	0	0	0/1	NA	NA	NA	1/0
117-81-7	Diethylhexyl phthalate	A	1	>50%	0.92	0.83	1	0	0	0	0	0	0	0	NA	0/1	NA	NA	NA
66230-04-4	Esfenvalerate	A	1	>50%	0.92	0.83	1	0	0	0	0	0	0	0	NA	NA	NA	0/1	NA
122-14-5	Fenitrothion	A	1	>50%	0.92	0.83	1	1	1	0	0	1	1	1	NA	NA	NA	1/0	NA
330-55-2	Linuron	A	1	>50%	0.92	0.83	1	1	1	0	0	1	1	0	1/0	1/0	NA	9/0	1/0
72-43-5	Methoxychlor	A	1	>50%	0.92	0.83	1	1	1	0	0	1	0	0	NA	1/0	NA	NA	0/1
52645-53-1	Permethrin	A	1	>50%	0.92	0.83	1	0	0	0	0	0	0	0	NA	NA	NA	0/2	NA
67747-09-5	Prochloraz	A	1	>50%	0.92	0.83	1	0	1	0	0	0	1	0	NA	NA	1/0	NA	NA
7696-12-0	Tetramethrin	A	1	>50%	0.92	0.83	1	0	0	0	0	0	0	0	NA	NA	NA	0/1	NA
3380-34-5	Triclosan	A	1	>50%	0.92	0.83	1	0	0	0	0	0	1	0	NA	NA	NA	NA	0/1
50471-44-8	Vinclozolin	A	1	>50%	0.92	0.83	1	1	1	0	0	1	1	0	1/0	1/0	1/0	4/0	1/0

[a] 1 indicates positive result, 0 indicates negative result

[b] (# of positive reports/# number of negative reports)

[c] Conflicting pubertal studies were ignored (See methods)

[d] Additional positives from other assays required for positive call (See methods)

Supplemental Material, Table S3: Chemical Results Overlapping HTS-A and Guideline-A Studies

Appendix D: Links to Additional Supplemental Data Files

Additional supplemental files can be found at the U.S. EPA's ToxCast webpage. The following supplemental files can be obtained under the download column for "Rotroff et al (2012), Using In Vitro High-throughput Screening Assays to Identify Potential Endocrine Disrupting Chemicals, Environmental Health Perspectives" at **http://epa.gov/ncct/toxcast/data.html**.

Upon downloading the .ZIP file, the 5 supplemental data files will be decompressed:

Supplemental_File_1.csv: File Containing ToxCast Data Used for Analysis

Supplemental_File_2.txt: Citations for Guideline and Non-Guideline Studies

Supplemental_File_3.csv: File Containing Guideline and Non-Guideline Data Used for Analysis

Supplemental_File_4.csv: Guideline Model Results

Supplemental_File_5.csv: Non-Guideline Model Results

References

Huang R, Xia M, Cho MH, Sakamuru S, Shinn P, Houck KA, et al. 2011. Chemical genomics profiling of environmental chemical modulation of human nuclear receptors. Environ Health Perspect. 2011 Aug;119(8):1142-8.

Judson RS, Martin MT, Reif DM, Houck KA, Knudsen TB, Rotroff DM, et al. 2010. Analysis of eight oil spill dispersants using rapid, in vitro tests for endocrine and other biological activity. Environ Sci Technol 44(15):5979-5985.

Kojima H, Katsura E, Takeuchi S, Niiyama K, Kobayashi K. 2004. Screening for estrogen and androgen receptor activities in 200 pesticides by in vitro reporter gene assays using Chinese hamster ovary cells. Environ Health Perspect. 112(5): 524-531.

Martin MT, Dix DJ, Judson RS, Kavlock RJ, Reif DM, Rotroff DM, et al. 2010. Impact of environmental chemicals on key transcription regulators and correlation to toxicity end points within EPA's ToxCast program. Chem Res Toxicol. 2010 Mar 15;23(3):578-90.

NICEATM / ICCVAM. 2011. NICEATM Draft ED BRD: BG1Luc ER TA Test Method – Section 3.0. http://iccvam.niehs.nih.gov/methods/endocrine/BG1Luc/Section3-24Jan2011.pdf. accessed 21 March 2011].

Nishihara T, Nishikawa J, Kanayama T, Dakeyama F, Saito K, Imagawa M, et al. 2000. Estrogenic Activities of 517 Chemicals by Yeast Two-Hybrid Assay. Journal of Health Science. 46(4): 282-298.

Vinggaard AM, Breinholt V, Larsen JC. 1999. Screening of selected pesticides for oestrogen receptor activation in vitro. Food Addit Contam. 16(12):533-542.
